# Why nanny statism matters: evidence from the first wave of COVID-19

**DOI:** 10.1186/s12889-024-19477-8

**Published:** 2024-07-23

**Authors:** Fabrizio Carmignani

**Affiliations:** School of Business, University of Southern Queensland, Toowoomba, Australia

**Keywords:** Paternalism, Nanny state, COVID-19, Public health

## Abstract

**Background:**

A nanny state imposes restrictions on people’s liberty and freedom of action in order to advance their interest and welfare. The extent to which this is desirable, or even ethically acceptable, is debated in the literature. This paper formulates and tests the following hypothesis: the more of a nanny a state has been in the past, the more likely it is that the incumbent government will respond to a new, unknown threat with interventions of a paternalist nature, irrespective of other factors that might contribute to shaping government’s response. This hypothesis is then taken to the data using the first wave of COVID-19 as an empirical test.

**Methods:**

Data are collected from secondary sources for a sample of 99 countries. Nanny statism is measured by the number of paternalist laws and regulations adopted by a country in the past. The response to COVID is proxied by the time of adoption of control and containment measures and their stringency. The public health outcome is measured by the COVID-19 death toll at the end of June 2020. These variables, plus several controls, are then used to estimate a set of linear and probit regressions and a proportional hazard model of the timing of adoption of control and containment measures.

**Results:**

An increase in nanny statism by 0.1 (on a scale from 0 to 10) on average increases the probability of adoption of control and containment measures by 0.077 (i.e. 7.7 percentage points). The central tenement of the hypothesis is therefore consistent with the empirical evidence. The linear and probit regressions also show that there is no evidence of a significant effect of nanny statism on the stringency of the measures adopted. Irrespective of stringency, however, early adoption of control and containment measures is found to reduce the death toll of COVID-19 in the first half of 2020: an increase in nanny statism by 0.1 reduces the COVID death toll by approximately 7%.

**Conclusions:**

A tradition of nanny statism potentially leads to a more timely and effective public policy response to a new, unknown crisis. Further tests of the hypothesis should look at the relationship between nanny statism and public health outcomes from natural disasters.

**Supplementary Information:**

The online version contains supplementary material available at 10.1186/s12889-024-19477-8.

## Background

Like parents with their children, a paternalist government imposes restrictions on people’s liberty and freedom of action in order to advance their interest and welfare. Examples include laws that require bikers to wear a helmet, motor vehicle drivers and passengers to use a seatbelt, producers to apply warning labels on alcohol or tobacco products, and norms that ban or restrict smoking, prevent the advertisement of certain foods, and limit the sale and consumption of alcohol in public places. While most countries in the world experience at least some degree of government paternalism, the debate on its merits (i.e. benefits and costs) and reason for existence is still quite vigorous. On the one hand, supporters argue that, in many instances, governments have more information and experience than citizens and hence are in a better position to determine what is best for them. This justifies interventions that, by affecting individual’s choices, improve societal health outcomes and wellbeing. On the other hand, opposers claim that this improvement is likely to be small and, in any case, does not justify intruding upon one’s autonomy and decision-making. The expression “nanny state” is sometimes used, with a negative connotation, to denote an overreaching, overprotective government.

This paper contributes to the debate by offering new evidence that nanny states have positive effects. However, rather than looking at how specific paternalist norms directly impact on corresponding behaviours or health outcomes (e.g. whether smoking bans reduce the incidence of smoking and/or the prevalence of respiratory and cardiovascular diseases), the paper argues a different, more general case. In countries with a tradition of stronger paternalism, the political and cultural environment should be in general more receptive to the idea that the government “knows better”. This then translates into a more favourable attitude from the public towards the imposition of regulations and restrictions, especially when the community faces new and unknown threats to public health. As a result, faster and more effective responses to these threats are undertaken, which generates broader benefits, at least to the extent that the government does know better in the first place. In short, the paper advances the hypothesis that the more of a nanny the state has been in the past, the more likely and faster the adoption of paternalist responses to a new crisis will be (with the corollary that this should then lead to better public health outcomes).

The COVID-19 pandemic provides an opportunity to test the hypothesis. The fast spreading of COVID-19 in early 2020 was accompanied by a general sense of uncertainty about the nature of the disease, its effects, and how to stop contagion. It was, in this respect, a health crisis that took the population by surprise. In the absence of a vaccine, the general recommendation was to avoid contact among individuals. But in heavily populated areas, this could only be achieved through control and containment measures (CCM), such as stay home orders, closing of school and offices, and other restrictions on people’s movement. Many countries ended up adopting this type of measures in 2020 but did so at different points in time and with different intensity. These differences allow for a test of the general hypothesis of the paper: countries where the state was already more of a nanny should have been the first ones to adopt CCM, after controlling for other factors that could explain adoption (or lack thereof).

The empirical analysis, based on a large sample of countries observed over the first six months of 2020, supports the hypothesis. The strength of the nanny state in each country is measured by the number of paternalist laws and regulations that were adopted before 2020. Higher values of this index significantly increase the probability of a country adopting some CCM as early as the beginning of March 2020, even though no COVID-related casualty had yet been reported in that country. The index however does not seem to explain the depth of stringency of CCM. In other words, one can think of government’s decision as consisting of two stages: (i) whether to impose any measures to control and contain the disease and (ii) how far to push these measures. A tradition of stronger paternalism affects the first stage more than the second one. However, the paper also provides evidence that the total COVID death toll by end of June 2020 was higher in countries that had not adopted any CCM early on, irrespective of how stringent these measures were. So, by making the adoption of at least some degree of containment and control more likely, nanny statism reduced the death count and, in this sense, contributed to a better health outcome for the entire population.

### Views on paternalism and the nanny state

The rationale for government paternalism can be laid out in terms of information asymmetries between government and individuals. When there is a body of rigorous evidence supporting a course of action that individuals would select for themselves if they had adequate information or experience, then a form a paternalism that leads to this course of action may be considered to improve the wellbeing of individuals as determined by the individuals themselves [1]. The fundamental question then becomes the extent to which government can (legally and ethically) impose a course of action to its citizens. In this respect, while coercion is generally undesirable, feasible regulations that do not limit important freedoms and whose costs and risk do not outweigh their benefits may be justifiable, again under the presumption that effectively the government knows better than its citizens [13]. In this sense, paternalism aligns with the notion of nudging used in behavioural economics [23, 28]. In arguing the case for (some degree of) nanny statism, the experience of the tobacco industry can be used to show how state intervention and regulation is a precursor to informed choice [15]. In the presence of strong commercial interests that can effectively distort the narrative around a particular type of product and its health effects, paternalism provides individuals with liberties of which they could be otherwise deprived and ultimately increases societal well-being above and beyond potential economic costs [14, 31]. At the same time, the literature [3, 10, 20] has also discussed the question of reasonable limitations of paternalism.

The case against paternalism builds on the view that the evidence of benefits for individuals and community is far from established. Clearly, in the absence of significant benefits, the costs of interventions that restrict individual’s autonomy and prevents them from making their own decisions would make paternalism unjustifiable and unacceptable [27]. To put it slightly differently, who says that the government knows better? The literature has looked at this question from two angles. First, a voluminous body of research has tried to estimate the direct impact of certain regulations on the specific outcomes they are meant to improve, e.g. whether tobacco control policies impact on smoking prevalence [4, 8] or whether mandatory helmet legislation affects fatal road traffic injuries [22]. While, on balance, there is some evidence of the effect of regulations on specific health outcomes, there is still substantial debate on the size of this effect and the other conditions and factors that might affect its strength (e.g. exact type of regulation or intervention, underlying socio-economic environment, etc…). Second, building on the idea that the acceptability of interventions is both a critical element of their effective implementation and an indicator of the degree to which people do believe that the government has better information, some new research investigates public’s perceptions of regulations and nanny statism or paternalism more generally [11, 16, 17, 19]. Again, results are qualified in a number of ways, but there seems to be broader acceptance for interventions that require higher levels of personal responsibility.

### Hypothesis

To further inform the debate, this paper formulates a new hypothesis concerning the benefits of nanny statism. To illustrate the hypothesis, consider a situation where a new threat to public health arises. This new threat is little known to the public (e.g. differently from what, it can be argued, tobacco or alcohol now are), so that effectively people’s information on how to deal with it is largely incomplete. The imposition of regulations and interventions to address the crisis might be complicated by the high degree of incompleteness of information. In fact, some individuals might think that since the challenge is so new and unknown, then nobody has better information than anybody else. These individuals will be more likely to oppose any sort of interventions enacted by the government. At the opposite end, other individuals will acknowledge that since they know so little about the threat, then it is in their own interest to be guided by the government, as if the government was their nanny. These different attitudes are likely to be driven by history. In countries with a tradition of paternalism, people should be more used to government intervention, and hence more inclined to acknowledge its value and usefulness. This in turn should make them more likely to accept and follow new regulations in the face of the unknown new threat. Conversely, a lack of a tradition of paternalism suggests that people would be less receptive to the idea that the government knows better, even in the face of an unknown new threat. Note that in this argument, the reasons why a country has or does not have a tradition of paternalism are substantially irrelevant. This tradition emerges over time as subsequent governments adopt a wider range of regulations covering more and more aspects of public health and individual choices. However, what matters for the hypothesis is how the tradition of paternalism (or lack thereof) translates into people being more or less used to having a nanny state that decides what is good for them.

The argument lends itself to a straightforward empirical test: the likelihood of adoption of new regulations in response to a relatively unknown threat should be higher in countries that have a stronger tradition of paternalism. To implement this test, one needs (i) a suitable empirical measure to capture the extent of paternalist tradition in a country and (ii) a suitable “threat” to which different governments responded in different ways. The first point is discussed in the next section. The rest of this section considers the second point.

### COVID-19 responses as a suitable test

The COVID-19 crisis provides a suitable scenario to test the hypothesis of this paper. Its emergence was sudden and confusing, at least to laypeople, due to the perceived seriousness of the illness, the proliferation of information from multiple sources, the difficulty in deciphering fact from fiction, and the lack of a convincing therapy, which arguably made COVID-19 look like a new disease despite its relations to other respiratory illnesses that had had limited spread in the past. Indeed, studies report significant evidence of pandemic-related anxiety among the public through the first wave of the disease [21]. As it became clear that the disease and its death toll could not be contained to China, governments worldwide started to consider preventive interventions. In the absence of a vaccine, these included the adoption of respiratory masks, closures of public offices and schools, and lockdowns, i.e. measures with a significant cost in terms of both individual freedom of movement and likely impact on the economy.

In all these respects, COVID-19 was a new threat to public health and the available policy responses, at least in the first months of 2020, involved paternalist interventions. The hypothesis is that countries with a stronger tradition of nanny statism would be more likely to adopt these paternalist interventions earlier and (possibly) more intensively than other countries. The test of the hypothesis therefore involves looking at the likelihood, timing, and intensity of adoption of CCM in early 2020.

### Adoption and stringency of CCM in the first half of 2020

In this paper, the stringency of CCM is measured by the index described in [12] and publicly available from The Oxford COVID-19 Government Response Tracker (OxCGRT) at www.bsg.ox.ac.uk/covidtracker. OxCGRT used a team of over 1500 trained volunteers worldwide to gather information on the strictness of the policies implemented by governments. This information was collected through government websites and official news reports, assessed, and interpreted in a standardised system that allows for cross-country comparability. The bulk of the data are provided in the form of categorical ordinal indicators that can then be aggregated to form comprehensive indices. The specific version of the stringency index used in this paper combines indicators that capture the following government interventions: school closures, workplace closures, cancellation of public events, restrictions on public gatherings, closures of public transport, stay-at-home requirements, public information campaigns, restriction on internal movements, and international travel controls. The index ranges from 0 (no measures implemented) to 100 (maximum stringency).

COVID peaked at different times in different countries through 2020 and 2021. However, many countries did experience an initial wave of COVID in the first half of 2020. In fact, all of the 158 countries for which data are available eventually adopted some degree of CCM by June 2020. The timing of adoption and the stringency of the measures varied across countries. For instance, as of end of June 2020, 25% of countries had a stringency index greater than 77.8 on a scale from 0 to 100, but another 25% of countries had a stringency index lower than 49.1. As of 1 March 2022, 50 countries (i.e. close to 1/3 of the total) had not yet adopted any measures. The average stringency was “only” 11.4, but some countries had already escalated stringency to 30 and a few even as high as 60. In comparison, by end of June 2022, the average level of stringency in the full sample was 62.5 and only 5% of countries had a stringency lower than 25. These cross-country differences in the timing of adoption and stringency of CCM provide the statistical variation required for the test of the hypothesis. In so doing, the paper also contributes to the growing literature on the adoption of COVID-19 control and containment measures [2, 18, 24, 29]. This literature however does not investigate the role that a tradition of paternalism has on the adoption of measures.

It should be noted that for CCM stringency to be a suitable dependent variable in the test, it is not strictly necessary that it had a positive effect on public health outcomes. In fact, the point of the test is to see whether a tradition of paternalism facilitates the adoption of new paternalist measures as a new threat emerges. Whether these measures improved public health is at best a corollary of the hypothesis. Nevertheless, since the very notion of paternalism finds its justification in the improvement of health outcomes for the community, it is important to assess if the adoption of CCM helped reduce morbidity and mortality in the first wave. Section 3 will present some evidence of a negative and statistically significant effect of CCM adoption on the death toll from COVID as of June 2020. This finding is consistent with other results previously reported in the literature [6, 9, 30].

## Methods

### Regression models

The test of the hypothesis argued in the previous section boils down to estimating the effect of a measure of nanny statism on the index of stringency of CCM. In its simplest form, this requires estimating the parameters of the following equation:

1 $${y}_{i}={\varvec{z}}_{i}^{\varvec{{\prime\:}}}\varvec{\gamma\:}+\beta\:{x}_{i}+{\epsilon\:}_{i}$$

Where *i* denotes a generic country, $\:y$ is the stringency index introduced in the previous section ([12]), $\:x$ is the measure of nanny statism, $\:\mathbf{z}$ denotes a set of other regressors (control variables), including a constant term, $\:\varvec{\gamma\:}$ and $\:\beta\:$ are the parameters to be estimated, and ϵ is a random disturbance. In particular, the testable implication of the hypothesis is that the estimated $\:\beta\:$ should be statistically greater than zero, meaning that a stronger tradition of nanny statism increases the stringency of the CCM adopted by the government.

For an effective statistical representation of the hypothesis formulated in Sect. 3, the dependent variable $\:y$ should be measured at a sufficiently early stage in 2020, so to fully capture the idea of a “new” threat. This would also ensure that empirical findings are not driven by a domino effect whereby a country adopts CCM only because other countries do so. There is, of course, no unambiguous way to determine when to measure $\:y$. However, considering that at global level the death toll from COVID started to increase rapidly during March 2020 and that by 1 April 2020 all countries had adopted at least some CCM, the beginning of March 2020 seems like a reasonable choice of when to measure $\:y$ .

**Fig. 1:**
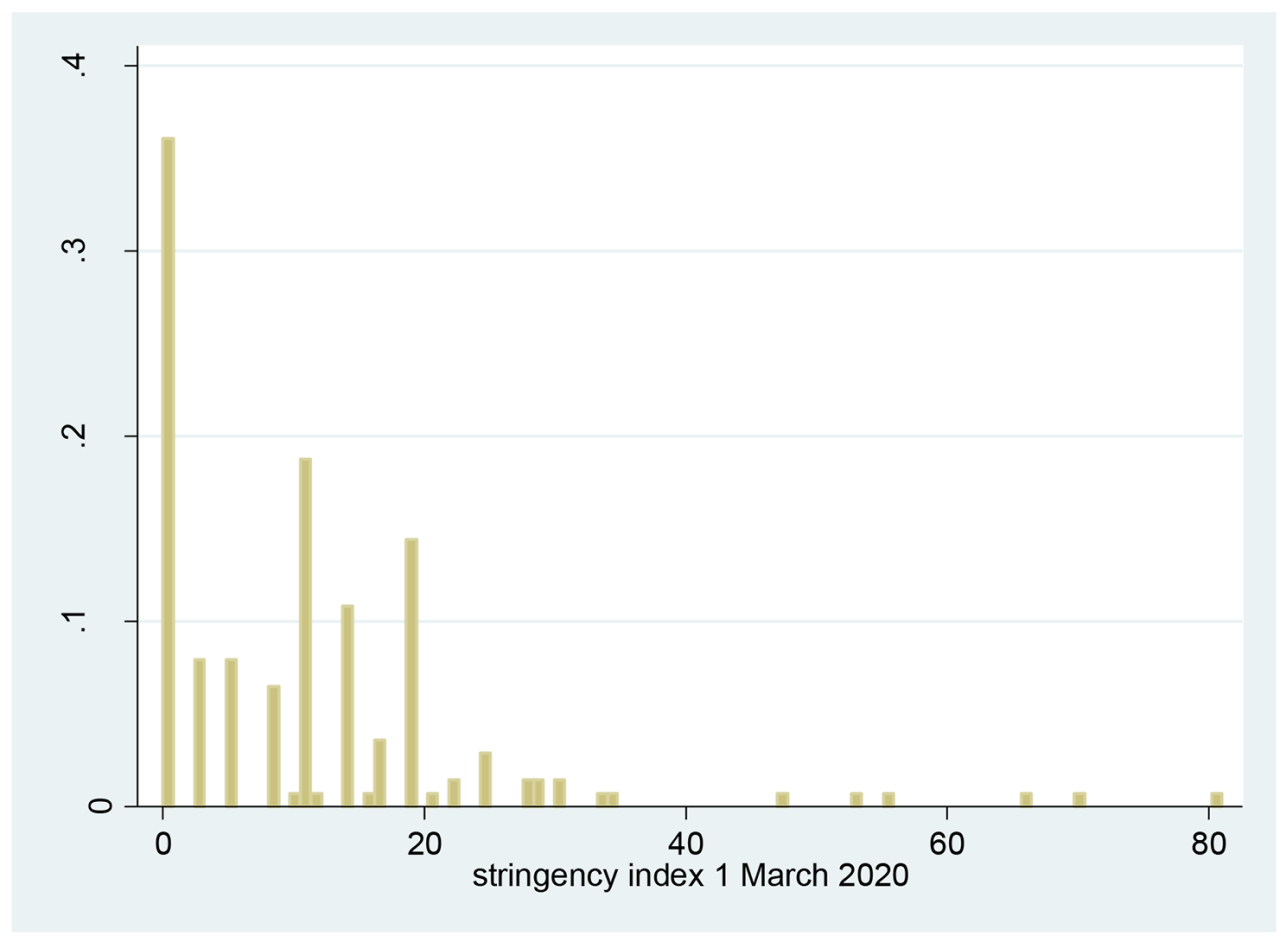
Distribution of CCM stringency index

Figure 1 plots the distribution of the stringency index as of 1 March 2020, i.e. the dependent variable $\:y$. As can be seen, a significant proportion of the variation across countries stems from the fact that many countries had in fact a stringency index of 0, meaning that they had not yet adopted any responses to COVID-19. This particular distribution of the dependent variable suggests that the observed outcome $\:y$ can be modelled as a two-step process: (i) the decision whether or not to adopt any CCM, and (ii) given the decision to adopt some CCM, how stringent these should be.

The first step involves a binary outcome, which can be modelled as follows. Let $\:{d}_{i}$ be a dummy variable that takes value 1 if the stringency index is positive and 0 if the stringency index is zero. Using the same notation as in Eq. (1), the conditional probability of $\:{d}_{i}=1$ is:

2 $${\:\:\:\:Pr}\left({d}_{i}=1|\varvec{z},\beta\:\right)=F({\varvec{z}}_{i}^{\varvec{{\prime\:}}}\varvec{\gamma\:}+\beta\:{x}_{i})$$

Where $$F(\cdot)$$ is a specified parametric function. A possible approach to estimation is to just assume $$\:F\left({\varvec{z}}_{i}^{\varvec{{\prime\:}}}\varvec{\gamma\:}+\beta\:{x}_{i}\right)=$$$$\:{\varvec{z}}_{i}^{\varvec{{\prime\:}}}\varvec{\gamma\:}+\beta\:{x}_{i}$$, which generates a liner probability model equivalent to a linear regression model like Eq. (1). In this case, however, the predicted probability generated from the estimation of the model is not bound between 0 and 1. A more common approach is to specify a probit model where $${F\:}\left(\cdot\right)={\Phi\:}\left(\cdot\right)$$ and $$\:{\Phi\:}\left(\cdot\right)$$ is the standard normal cumulative distribution function. Some evidence from both these approaches will be presented in this paper. The second step can then still be estimated as a linear regression using Eq. (1), but only using the sample of non-zero observations; i.e. the sample of countries for which $$\:{y}_{i}>0$$.

### Survival analysis

To further explore the impact of nanny statism on the timing of adoption of CCM, the paper also makes use of survival analysis. In a nutshell, the adoption of CCM is modelled as a stochastic process that starts on 1 January and ends on the first day when the stringency index is not zero. The probability that adoption will occur at a given point in time *t*, conditional on not having occurred until time *t*, is then defined as:

3 $$\:h\left(t\right)=\underset{\varDelta\:t\to\:0}{\text{lim}}\frac{P[t<T<t+\varDelta\:t|T\ge\:t]}{\varDelta\:t}$$

Where $\:T$ denotes the end of the stochastic process and $\:P[\cdot]$ is the notation for the conditional probability. Equation (3) is known has the hazard function. Under the assumption that nanny statism and other controls have a proportional impact on the hazard function, the estimating model takes the form:

4 $$\:h\left(t,x,\varvec{z}\right)=\text{e}\text{x}\text{p}(\beta\:{x}_{i}+{\varvec{z}}_{i}^{\varvec{{\prime\:}}}\varvec{\gamma\:}){h}_{0}\left(t\right)$$

Where *i* is a generic country, $\:x$, $\:\varvec{z}$, $\:\beta\:$, and $\:\varvec{\gamma\:}$ are the same as in Eqs. (1) and (2), $\:\text{exp}\left(\cdot\right)$ is the notation for the exponential function, and $\:{h}_{0}\left(t\right)$ is the baseline hazard function, i.e. a baseline function defined for the case where the expected value of $\:x$ and $\:\varvec{z}$ is zero.

Equation (4) is estimated using a flexible semi-parametric approach that does not require the choice of a specific functional form for $\:{h}_{0}\left(t\right)$, see [7]. The effect of nanny statism can then be expressed in terms of the hazard ratio, i.e. the proportional change in the hazard function for a one-unit change in the nanny state index. As discussed below, the nanny state index is defined as a proportion, and hence it is bounded between 0 and 1. However, to facilitate the interpretation of the results from the survival analysis, it will be rescaled as a percentage (i.e. bounded between 0 and 100). A hazard ratio of 1 means that nanny state does not affect the hazard function. A hazard ratio significantly greater than 1 means that an increase in the nanny state index increases the hazard and hence reduces the time to adoption of CCM; vice-versa if the hazard ratio is significantly lower than 1.

A further methodological point concerns the case of a country that has not adopted any stringency measure by 1 March 2020, i.e. $\:{T}_{i}>61\:$(61 being the number of days from 1 January to 1 March). A first option is to simply count days past 1 March 2020. The second option is to stop counting at 1 March 2020 and treat the observation as censored. The second option seems to be more consistent with the econometric set-up used for Eqs. (1) and (2). However, theoretically there is no ground to prefer one option over the other, hence results obtained from both options will be presented in the next section.

Lastly, one could think of alternative set-ups for the survival analysis, particularly with respect to the start and end point of the stochastic process. For instance, rather than 1 January 2020, the starting point could be the day of the first reported COVID case (or death). Similarly, the end point could be set as the day when the stringency index achieves a certain (pre-determined) value or its maximum value. Clearly, different choices of start and end point would correspond to slightly different hypotheses on the role of nanny statism and require different treatments for censored observations. For the purpose of this paper, it seems that starting the process as of 1 January and ending it on the day of adoption is the best way to capture the hypothesis formulated in Sect. 1.

### Measuring nanny statism

The empirical definition of variable $\:x$, i.e. nanny statism, is clearly central to the implementation of the statistical test. [26] proposes and index that measures legislative restrictions on eating, drinking, smoking, and vaping in Europe. For each of three main categories (alcohol, nicotine, and diet), this index assigns points based on a variety of criteria that reflect the extent to which consumers are negatively affected by paternalistic policies. Higher values of the index denote less freedom for consumers and hence are indicative of a more pervasive nanny state. In the 2021 league table, Norway was ranked as the least free country, with a total score of 51.5; Germany was instead the freest country, with an index of 10.7.

While this index is certainly interesting, its use in this paper would present two complications. First, from a methodological perspective, the index is constructed from a rather complex system of weighting and scoring across a variety of criteria based, in some cases, on country expert’s subjective assessment of the depth and extent of relevant laws. Second, and more importantly, the index covers only 30 countries, i.e. too small of a sample for a cross sectional analysis.

In view of these complications, this paper measures the nanny state in a simpler way, as follows. The WHO Global Health Data Repository provides information on laws and regulations that affect or substitute for personal choice in a global cross-section of countries. Based on this information, 21 categories are identified, each category representing a particular area of potential government intervention (e.g. use of helmets, vehicle safety standards, marketing of foods for children, warning labels on certain products, etc…). For each category, a binary variable is then coded as 0 if no law or regulation is adopted by a country in relation to that category and 1 if a low or regulation is in existence as of 2019. The nanny state index for each country is then simply equal to the proportion of ones on the total of 21 categories. An “extended” index is also calculated using four additional categories. For a few countries, information is not available for all categories, so that the binary variable can only be coded for a subset of the total set of 21 categories. In these cases, the nanny state index is equal to the proportion of ones on the number of categories in the subset. Note that since the index is constructed based on laws and regulations that were in place as of 2019, it is pre-determined relative to the adoption of CCM in 2020. Further details on how the nanny state index is constructed can be found in the Supplementary Materials.

The index is theoretically bound between 0 and 1. In practice, only Haiti and Liberia have a perfect zero score, while Portugal has the maximum score (0.95). The average across all the 179 countries for which information is available is 0.46, with a standard deviation of 0.22. A cursory look at the ranking of countries suggests that very low values of the index tend to be observed in poor countries with generally weak institutions. In fact, the bilateral correlation coefficient of the index with GDP per-capita and government effectiveness (see definition below) is 0.49 and 0.6, respectively. While clearly not perfect, this positive and significant correlation suggests that it is important to control for the effect of GDP per-capita and government effectiveness in assessing the effect of the nanny state on CCM. On the other hand, the bilateral correlation of the nanny state index with quality of the polity and ideological orientation of the government is rather low (0.19 for polity and − 0.15 for ideology).

### Regressors and sample for estimation

The vector of regressors ***z*** in Eqs. (1) and (2) includes variables that can be arguably expected to affect the adoption and stringency of CCM. Based on the existing literature, two factors that seem to be particularly important in explaining adoption and stringency of CCM are government’s capacity and perceived level risk of exposure. As a pragmatic approach to estimation, vector ***z*** is initially specified parsimoniously using a small number of empirical variables to capture each of these two factors. Some sensitivity analysis is then conducted to see whether the addition of more variables to vector ***z*** affects the estimate of parameter $\:\beta\:$ (which is ultimately the result of interest for this paper).

In the baseline specification, government’s capacity is captured by the level of GDP per-capita and an index of government effectiveness available from the Worldwide Governance Indicators Project. The former is a general proxy for spending capacity and stage of economic development. The latter instead picks the quality of public service, capacity of civil service and its independence from political pressures, and the general quality of policy formulation. In theory, greater spending capacity and stronger effectiveness should lead to faster responses. The perceived risk of exposure is represented by two variables related to the demographics of a country. In fact, it became clear in the early stages of the pandemic that the elderly population was at greater risk of contagion and that the disease would spread more quickly in crowded environments. The variables used to proxy for these effects are the elderly share of total population and the average population density in the country.

The additional variables used for the sensitivity analysis are an index of quality of the polity to allow for the effect of democracy, a measure of the political ideology of the incumbent government, the prevalence of obesity, diabetes, and tuberculosis to account for the increased risk due to co-morbidities, and the geographical distance between the country and China. This latter variable accounts for the fact that in the very initial stages, the disease was mostly prevalent in China and there could have been a belief that countries nearer to China could be first affected by the wave of contagion. All these variables, including those in the baseline specification, are measured as of 2019 or prior to 2020. Variables definition, sources, and summary statistics are reported in the Supplementary Materials.

It would be reasonable to expect the adoption and stringency of CCM to be affected by the evolution of the pandemic, both domestically and internationally. However, the evolution of the pandemic is also affected by CCM, so that an issue of endogeneity would arise in the estimation of Eqs. (1), (2) and (4). This issue is dealt with in three ways. First, the sample for estimation is restricted to countries that had not yet reported a COVID death as of 1 March 2020. Second, to account for the incidence of the pandemic, the number of COVID cases as of end of February 2020 will be included in the sensitivity analysis. Third, spatial variables that capture the intensity of the pandemic and government response in neighbouring countries will also be added to the set of controls in some of the sensitivity checks.

Once restricted to countries that had not yet reported a COVID death as of 1 March 2020, the potential size of the sample is reduced to 99 countries. However, some robustness checks using the full sample of all countries for which data are available will also be presented. It is worth stressing that not all variables are available for all countries. This means that, depending on which variables are included, the total number of observations may drop below 99. The actual sample size is reported at the bottom of each table in the next section.

## Results

### Preliminaries: stringency of control and containment measures (CCM) and COVID-19 deaths

As already discussed in Sect. 1, whether CCM effectively reduce COVID-related deaths or not is not strictly part of the hypothesis formulated in this paper. Still, the whole idea of paternalism and nanny statism is founded on the presumption that government intervention improves public health outcomes. Therefore, before presenting the results of the estimation of Eqs. (1), (2) and (4), it is important to check if CCM have any impact on the COVID-19 death toll. It should be noted that the death toll is only one of several possible health outcomes (including mental health outcomes) that could be affected by CCM. While it is not the purpose of the paper to provide a full investigation of all other public health outcomes, this could be the scope of future research.

Table 1 reports the estimated coefficients of a linear regression of the COVID death toll as of June 2020 (which is here used to mark the end of the first global wave) on CCM stringency and a set of control variables. Stringency is captured by the dummy variable previously introduced, namely $\:{d}_{i}$ = 1 if the CCM stringency index is positive as of 1 March 2020 and 0 otherwise. The estimated coefficient of this variable therefore represents the effect on deaths due to adopting/not adopting some CCM (irrespective of stringent these CCM were). In column II of the table, however, the numerical index is used instead of the dummy variable; in this case the estimated coefficient of the index also captures the effect due to the actual stringency of the measures.

The negative sign of the coefficient of the dummy variable suggests that the early adoption of measures (that is, whether any measures were adopted or not by 1 March 2020) significantly reduced the cumulative death toll of the first wave. This finding is robust to the inclusion of a variety of controls to account for demographics (population density and age), government’s capacity (GDP per-capita, government effectiveness, quality of the polity), co-morbidities in the population (obesity, diabetes, tuberculosis) and health system’s capacity (hospital beds). However, in column II, the coefficient of the numerical index is statistically insignificant, meaning that beyond adoption, the intensity (or actual stringency) of the measures did not really have much impact on the death toll. Therefore, from a policy perspective, it was the early decision to adopt some CCM, rather than the actual stringency of the CCM themselves, that made a difference in terms of public health outcomes.

**Table 1:** The effect of CCM adoption and stringency on COIVD-19 death toll

	I	II	III	IV	V
Stringency dummy	− 0.730** (0.313)		− 0.700** (0.329)	− 0.768** (0.361)	− 0.685** (0.317)
Stringency index		− 0.028 (0.019)			
GDPpc	0.880*** (0.209)	0.849*** (0.223)	1.165*** (0.237)	0.945*** (0.252)	0.542 (0.403)
Population density	− 0.001 (0.001)	− 0.001 (0.001)	− 0.000 (0.001)	− 0.000 (0.001)	0.000 (0.001)
Elderly population share	0.130*** (0.029)	0.125*** (0.028)	0.090** (0.039)	0.170*** (0.042)	0.077* (0.042)
Government effectiveness	− 0.519 (0.324)	− 0.446 (0.365)	− 0.838** (0.412)	− 0.616 (0.399)	− 0.367 (0.478)
Polity			0.047 (0.034)		
Hospital beds				− 0.172 (0.126)	
Obesity					0.043* (0.022)
Diabetes					− 0.042 (0.047)
TBC					0.000 (0.001)
Constant	-6.167*** (1.901)	-6.109*** (2.105)	-8.736*** (2.183)	-6.599 (2.308)	-4.509 (2.978)
Observations	90	90	82	72	88

Note: Dependent variable is the number of COVID deaths per million people. Robust standard errors are reported in brackets. The sample excludes countries where at least one COVID death had been reported by the end of February 2020. *,*,*** denote statistical significance at the 10%, 5%, and 1% confidence level respectively

The finding from Table 1 suggests that it might be more relevant to focus the test of the hypothesis on Eq. (2) rather than Eq. (1). Equation (2) tests the effect of nanny statism on the binary choice “adopt/not to adopt”, which – as just seen - is what actually made a difference for the public health outcome. In Eq. (1), instead, the dependent variable mixes the binary choice to adopt or not adopt with the second stage choice of how stringent measures should be, which is less significant in terms of public health outcome.

### Baseline model estimates

Table 2 presents the core evidence from the empirical test of the hypothesis. All the regressions in this table are estimated using the more parsimonious specification of vector ***z***. To start with, column I shows estimates from Eq. (2) using a linear probability model. The positive and significant coefficient of the nanny state index means that the likelihood of adopting some CCM by 1 March 2020 is higher in countries where the state had been more of a nanny prior to 2020. The hypothesis put forward in Sect. 2 is therefore consistent with this evidence: in countries that have traditionally been more paternalist, governments are more likely to respond to a new threat with interventions that are of a paternalist nature. This finding is qualitatively unchanged when Eq. (2) is estimated as a probit (column II) and when the extended version of the nanny state index is used instead of the standard definition (columns III and IV).

**Table 2:** Baseline estimates of the effect of nanny statism on CCM adoption and stringency

	I Stringency Dummy	II Stringency Dummy	III Stringency Dummy	IV Stringency Dummy	V Stringency Index	VI Stringency Index > 0
Nanny State	0.745** (0.294)	2.297** (0.957)			7.247 (4.801)	-1.928 (4.513)
Nanny State extended			0.687** (0.298)	2.014** (0.913)		
GDPpc	0.053 (0.063)	0.182 (0.197)	0.056 (0.062)	0.173 (0.189)	1.282 (0.995)	0.806 (0.966)
Population density	0.001** (0.000)	0.002 (0.001)	0.001** (0.000)	0.002* (0.001)	0.006* (0.003)	0.002 (0.003)
Elderly population share	− 0.015 (0.011)	− 0.052 (0.037)	− 0.017 (0.011)	− 0.052 (0.035)	− 0.432* (0.234)	− 0.312 (0.213)
Government effectiveness	− 0.028 (0.105)	− 0.129 (0.300)	− 0.034 (0.108)	− 0.128 (0.304)	1.730 (1.478)	3.547*** (1.257)
Constant	− 0.090 (0.589)	-2.013 (1.842)	− 0.092 (0.596)	-1.840 (1.754)	-3.546 (1.478)	8.787 (7.679)
Observations	99	99	99	99	99	65

Note: Dependent variable is the Stringency dummy in columns I, II, III and IV, the Stringency Index in column V, and the positive values of the Stringency Index in column VI. Estimation is by linear regression in Columns I, III, V and VI and by probit in Columns II and IV. Robust standard errors are reported in brackets. The sample excludes countries where at least one COVID death had been reported by the end of February 2020. *,*,*** denote statistical significance at the 10%, 5%, and 1% confidence level respectively

The probit estimates are particularly useful as they allow to calculate the predicted marginal effect of nanny statism on the probability of adopting CCM. The average marginal effect (from the estimate in Column II) is 0.77. To interpret this finding, remember that the nanny state index is bounded between 0 and 1. So, an increase in the index by 0.1 on average increases the probability of adoption by 0.077 (i.e. 7.7 percentage points). The marginal effect is however stronger at lower levels of the nanny state index. For instance, the predicted probability of adoption when the nanny state index is 0.1 (and the other regressors are at their mean values) is 38%. If the nanny state index increases to 0.2, then the predicted probability increases to 47%, i.e. an increase of 9 percentage points. Conversely, if the nanny stage index is 0.7, the predicted probability is 86% and a further increase of the index to 0.8 “only” raises the probability to 90%, i.e. an increase of 4 percentage points.

The last two columns of Table 2 present estimates of Eq. (1), where the dependent variable is the full numerical stringency index, i.e. the index taking values from 0 to (theoretically) 100 instead of the dummy variable. In column V, the sample consists of all the 99 countries, therefore including those that had not yet adopted any measure by 1 March 2020. In column VI instead the sample only includes countries for which the stringency index was strictly positive as of 1 March 2020. In this respect, column VI can be seen as a representation of the second step in government’s decision-making process (that is, the decision of how stringent measures should be given that in the first step a decision has been made to adopt some measures). In both columns, the estimated coefficient of the nanny state index is largely insignificant, meaning that there is no evidence of any effect of nanny statism on the actual stringency of CCM, beyond adoption. Therefore, it looks like a tradition of paternalism is relevant in determining whether the government adopts some CCM, but not in determining the intensity of these measures once the decision to adopt them has been made. As previously noted, this result does not really invalidate the hypothesis of the paper.

Turning to the control variables in the regression, their statistical performance is generally rather weak. There is evidence of some demographic effect, even though the estimated coefficient of the share of elderly population is not consistent with a-priori expectations. Government effectiveness instead seems to be the main driver of the intensity of stringency once a decision to adopt some measures has been made. Because of this relatively poor statistical performance of the controls, it is important to check that the results concerning the nanny state index are robust to changes in the specification of vector ***z***.

### Robustness and sensitivity checks

The first five columns of Table 3 report a number of sensitivity checks. All these regressions are estimated using the probit parametrization for Eq. (2). In column I, the index of quality of the polity and the geographical distance of a country from China are added to the set of controls. The estimated coefficient of polity is statistically significant, suggesting that countries with a better polity (i.e. more democratic and with more checks and balances) are more likely to adopt CCM. This stands in contrast with the idea that these measures, being restrictive and limiting individual freedoms, would be more likely adopted by autocratic governments.

**Table 3:** Extended specification and sensitivity analysis of the effect of nanny statism on CCM

	I Stringency Dummy	II Stringency Dummy	III Stringency Dummy	IV Stringency Dummy	V Stringency Dummy	VI Deaths from COVID	VII Deaths from COVID
Nanny State	2.754*** (1.029)	2.940*** (1.111)	2.930* (1.713)	2.121** (0.838)	2.511** (1.035)	1.041 (1.070)	0.352 (1.094)
GDPpc	0.101 (0.218)	0.157 (0.276)	− 0.103 (0.479)	0.202 (0.001)	0.085 (0.222)	0.883*** (0.213)	0.561 (0.409)
Population density	0.002 (0.001)	0.002 (0.001)	0.002* (0.001)	0.001 (0.001)	0.001 (0.001)	− 0.000 (0.001)	0.000 (0.001)
Elderly population share	− 0.084** (0.042)	− 0.062 (0.047)	− 0.119* (0.068)	− 0.070** (0.029)	− 0.064 (0.041)	0.119*** (0.030)	0.075* (0.042)
Government effectiveness	− 0.185 (0.352)	− 0.136 (0.366)	0.069 (0.725)	− 0.145 (0.227)	− 0.057 (0.368)	− 0.641* (0.362)	− 0.420 (0.508)
Polity	0.060** (0.030)	0.051 (0.032)	0.106 (0.010)	0.077*** (0.025)	0.058* (0.031)		
Distance from China	− 0.330 (0.256)	− 0.185 (0.270)	0.014 (0.650)	− 0.460** (0.232)	− 0.360 (0.221)		
Obesity		− 0.011 (0.010)					0.041* (0.023)
Diabetes		0.016 (0.053)					− 0.041 (0.047)
TBC		0.001 (0.001)					0.000 (0.002)
Political ideology			− 0.034 (0.243)				
COVID-19 reported cases as of end of February 2020					− 0.331 (0.221)		
Stringency Dummy						− 0.848** (0.362)	− 0.719** (0.344)
Constant	1.363 (3.140)	− 0.0366 (3.542)	− 0.007 (6.627)	1.915 (2.667)	1.904 (3.139)	-6.579*** (1.948)	-4.717 (3.050)
Observations	92	92	42	146	91	88	88

Note: Dependent variable is the Stringency dummy in columns I to V and the number of COVID deaths per million people in Columns VI and VII. Estimation is by probit in Columns I to V and by linear regression in Columns VI and VII. Robust standard errors are reported in brackets. The regression in Column IV is estimated on the full sample of all countries. Regressions in all other columns are estimated on the sample that excludes countries where at least one COVID death had been reported by the end of February 2020. *,*,*** denote statistical significance at the 10%, 5%, and 1% confidence level respectively

In column II, measures of prevalence of various co-morbidities are also included as controls, but their effect is largely insignificant. A possible explanation for this is that as the set of controls is expanded, the risk of multicollinearity increases. For instance, there is likely to be a correlation between demographics and co-morbidities and/or between GDP per-capita and co-morbidities. This multicollinearity then tends to reduce the precision of estimates, thus implying that most estimated coefficients do not pass the zero-restriction test.

In column III, the set of controls includes an index to capture the political ideology of the incumbent. This is defined as a trichotomous variables that takes values 0, 1, 2 depending on whether the government is left, centre or right. Unfortunately, this variable is available for only a subset of countries, so the total number of observations available for estimation drops to 42. The estimated coefficient is however not significant, meaning that right (or left) governments were no more or less likely to adopt CCM than left (or right) governments.

Column IV reports estimates of Eq. (2) based on the full sample of all countries (i.e. without excluding those that had already experienced a death as of 1 March 2020, but still excluding China). Note how the geographical distance from China now plays a significant role, i.e. countries nearer to China effectively were more likely to adopt CCM in the very early stages of the pandemic. Most likely, this is because in those early stages, the idea was that the pandemic would have spread progressively from what was consider ground zero outward to neighbouring countries first. In retrospect, we know that the contagion did not spread in that way. In fact, the second major cluster of contagion and deaths after China occurred in Italy. Lastly, in column V, the number of COVID cases at the end of February 2020 is added as a control. The risk of reverse causality is mitigated by the fact that there is likely a lag between CCM adoption and reduction in cases. Nevertheless, some caution should be used in interpreting the lack of statistical significance of the estimated coefficient of the COVID-cases variable.

Through all these sensitivity checks, the estimated coefficient of the nanny state index remains positive and statistically significant, therefore confirming the results from Table 2. Note that in the case of column III, the estimated coefficient is only significant at the 10% confidence level. This is because of the reduced number of observations in the sample, which in turn reduces the precision of all estimates.

Table A in the Supplementary Materials reports some further sensitivity analysis based on the estimation of Eq. (1), with the full numerical stringency index (that is, the index defined over the theoretical scale from 0 to 100) as the dependent variable. Confirming the findings from Table 2, the estimated coefficient of the stringency index is generally not statistically significant. This is therefore further evidence that nanny statism affects the adoption of CCM but not necessarily their intensity once adopted. The same Table A in the Supplementary Materials also shows that the estimates of the coefficient of the nanny state index are robust to the inclusion in Eqs. (1) and (2) of variables that capture country’s past history/experience with other communicable zoonotic diseases (SARS, MARS, Ebola). In fact, this type of past history/experience could make a country more likely to adopt CCM in response to COVID-19. However, the estimates indicate that this effect is not statistically significant.

### Residual effect of nanny statism on COVID-related deaths

So far, the estimates have shown that (i) nanny statism increases the probability of adopting some CCM as of 1 March 2020, and (ii) having some CCM in place as early as 1 March 2020 contributes to reducing the death toll from COVID-19 as of the end of June 2020. These results (especially the first one) provide some validation to the hypothesis formulated in this paper. There is however a tangential question that might be worth exploring, albeit briefly. Does nanny statism affect the death toll through some channels other than the adoption of CCM? In other words, having established that nanny statism contributes to public health by facilitating the adoption of CCM, is there any other mechanism linking nanny statism and the death toll? One can for instance argue that in countries where there is more paternalism, individuals could be better aware of health hazards, which in the case of COVID could have translated into individuals being more willing to take simple hygiene precautions that could have limited the spread of the disease.

To answer this question, the regression presented in Table 1 is re-estimated including the nanny state index as a regressor. If nanny statism only affects the death toll via its effect on the adoption of CCM, then the estimated coefficient of the stringency dummy variable should remain negative and significant (as it was in Table 1) and the estimated coefficient of the nanny state index should be insignificant. Conversely, if there is another channel of transmission, then the estimated coefficient of the nanny state index should be statistically different from zero. Results are reported in columns VI (baseline specification) and VII (extended specification) of Table 3. As can be seen, the estimated coefficient of the nanny state index is largely insignificant, meaning that nanny statism only affects the public health outcome via its effect on the adoption of CCM.

### Nanny statism and compliance with CCM

If citizens in a country with a tradition of paternalism are willing to accept new paternalist interventions, then it can be argued that they should also be prepared to comply with those interventions. Hence, nanny statism should increase the extent of compliance with paternalist regulations. The estimates in the top seven rows of Table 4 provide some evidence that this is indeed the case. The table reports the estimated coefficient of the nanny state index in a regression of the changes in community mobility relative to before the beginning of the pandemic (sourced from Google COVID-19 Community Mobility Trends). These changes are expressed in absolute values, so that higher values denote greater compliance. The set of controls includes the COVID death toll (community mobility is measured as of 1 April 2020 and the death toll is taken as of the preceding week), the stringency index, and different measures of trust sourced from the Integrated Values Survey and the World Values Survey. The reason for such a parsimonious set of controls is that other demographic and social characteristics of the population tend to be highly correlated with the death toll.

**Table 4:** Further evidence on the role of nanny statism on compliance and adoption of CCM

	Dependent variable	Estimated coefficient on nanny state index	Controls	Sample, number of obs.
I	Mobility	24.651*** (6.786)	COVID deaths, stringency index	Full, 123
II	Mobility	26.409*** (8.399)	COVID deaths	Full, 123
III	Mobility	34.783*** (9.048)	COVID deaths	Restricted, 77
IV	Mobility	23.490*** (7.379)	COVID deaths, stringency index	Restricted, 73
V	Mobility	26.867** (13.468)	COVID deaths, trust in people	Full, 55
VI	Mobility	28.614*** (7.582)	COVID deaths, stringency index, trust in people	Full, 54
VII	Mobility	28.855*** (7.742)	COVID deaths, stringency index, trust in people, trust*stringency index	Full, 54
VIII	Mobility	16.364* (9.658)	COVID deaths, stringency index, trust in government, trust*stringency index	Full, 67
IX	Mobility	18.425* (10.003)	COVID deaths, stringency index, trust in neighbours, trust*stringency index	Full, 66
X	Stringency dummy	2.447*** (0.953)	Baseline, CCM adopted in neighbour countries (binary)	Restricted, 99
XI	Stringency dummy	2.545*** (0.991)	Baseline, CCM adopted in neighbour countries (value)	Restricted, 99
XII	Stringency dummy	2.430** (0.983)	Baseline, COVID deaths in neighbour countries (binary)	Restricted, 99
XIII	Stringency dummy	2.327** (0.975)	Baseline, COVID deaths in neighbour countries (value)	Restricted, 99

Notes: : In rows I to IX estimation is by linear regression. The dependent variable is the index of mobility as of 1 April 2020; an increase in the index denotes greater compliance with CCM. In rows X to XIII estimation is by probit regression and the dependent variable takes value 1 if a country had adopted any CCM as of 1 March 2020. The full sample includes all countries for which data are available. The restricted sample includes only countries that had not yet reported a COVID death by end of February 2020. This restricted sample is used for consistency with the regressions reported in Tables 2 and 3. Robust standard errors are reported in brackets. *, **, *** denote statistical significance at 10%, 5%, and 1% confidence level, respectively

The estimated coefficient of the nanny state index is always positive and significant, meaning that nanny statism increases compliance. The level of statistical significance is somewhat reduced in some of the regressions that include trust variables. This could be due to some multicollinearity and/or the reduced size of the sample. The estimated coefficients of all the controls can be found in Table B of the Supplementary Materials. In general, both the total death toll and stringency increase compliance, as one would expect. The effect of trust instead is negative and/or statistically insignificant. Previous evidence from a sample of European regions finds a positive effect of trust in government on compliance, without however controlling for paternalism, see [5]. There is therefore scope for future research on the interactions between paternalism and trust and their joint effect on compliance.

These findings on compliance could explain why nanny statism increases the likelihood of early adoption of CCM but not their level of stringency. If people in more paternalist countries tend to comply more, then early adoption becomes more effective and hence there is no need to increase the stringency of measures. This would also explain why higher stringency does not necessarily reduce the death toll, as shown in Table 1. More work will be needed in the future to explore this point. Future work could also look at social learning effects that have a time dimension, e.g. whether some individuals become more compliant over time by observing the behaviour of other individuals. In a cross-sectional set-up, it is statistically challenging to test for this type of time effects. For instance, adding a lag of the dependent variable to the regression would change the interpretation of the model, as the estimated coefficients would capture the effect of variables (including nanny statism) on the change in compliance rather than its level. While a potentially interesting question, estimating the determinants of growth in compliance during COVID-19 goes beyond the scope of this paper.

### Spatial effects

The escalation of stringency and/or CVOID deaths in neighbouring countries in the first half of 2020 could have pushed domestic authorities to adopt CCM more rapidly or intensively [25]. To account for these effects, spatial variables are added to the controls in the probit regression of stringency. These spatial variables are (i) the average value of the stringency index in neighbouring countries as of 1 March 2020 and (ii) the total death toll in neighbouring countries as of 1 March 2020. Two binary variables are also coded: one takes value 1 if at least one neighbour adopted CCM by 1 March 2020 and the other takes value 1 if at least one neighbour had reported a COVID death by 1 March 2020. Results for the nanny state index are shown in the bottom four rows of Table 4; the full set of results is reported in the Table B of the Supplementary Materials. The estimated coefficient of the nanny state index remains positive and statistically significant. Instead, none of the estimated coefficients of the spatial variables turns out to be statistically different from zero.

### Survival analysis of the effects of nanny statism

The evidence from the survival analysis is summarised in Table 5. As discussed in the methodological section, results are presented in the form of the hazard ratio of the nanny state index (hazard ratios for the control variables are reported in Table C of the Supplementary Materials). The *p*-values (in brackets) are calculated for the null hypothesis that the hazard ratio is 1.

**Table 5:** The impact of nanny statism on the delay of CCM adoption

	I	II	III	IV	V
Hazard ratio for nanny state index (*p*-value)	1.028 (0.001)	1.025 (0.001)	1.024 (0.001)	1.014 (0.030)	1.013 (0.011)
Observations	98	98	98	152	152
Test of PH assumption (*p*-value)	3.97 (0.5534)	9.49 (0.0911)	…	5.91 (0.3152)	5.34 (0.3759)
Censoring	Yes	No	No	Yes	No
Sample	Restricted	Restricted	Restricted	Full	Full

Notes: The table reports the hazard ratios from the Cox Proportional Hazard model, except form Column III where hazard ratios are estimated from a parametric model based on the Weibull distribution. *P*-values for the test of the null hypothesis that the hazard ratio is 1 are reported in brackets. The test of the proportional hazard (PH) assumption is a chi-square test of the null hypothesis that the log hazard-ratio function is constant over time; that is, that the hazard ratio is constant over time. The table reports the value of the chi-square test statistic and the corresponding *p*-value in brackets. In some models, countries that had not yet adopted CCM as of 1 March 2020 are treated as censored observations (Columns I and IV). The restricted sample includes only countries that had not yet reported a COVID death by 1 March 2020. The full sample includes all countries for which data are available. The set of controls is the same as in Table 2

In column I, the survival model is estimated on the sample of countries that had not reported a COVID death as of 1 March 2020. Countries that had not adopted any stringency measures by 1 March 2020 are treated as censored observations. The hazard ratio is statistically greater than one, meaning that CCM were adopted earlier in countries with a stronger tradition of nanny statism. In column II, all observations are treated as uncensored, in column IV the sample includes all countries for which information is available, and in column V observations are uncensored and all countries are included in the sample. The hazard ratio of the nanny state index remains greater than one across all these permutations.

The assumption underlying the estimation of the survival model is that regressors have a proportional effect on the hazard function. If this assumption is correct, then the log of the hazard function must be constant over time. A test of this null hypothesis is provided in the table. It appears that the null hypothesis cannot be rejected at the 1% confidence level, except for the model in column II (where the hypothesis cannot be rejected at the 2% confidence level). The model in column II is also re-estimated parametrically using the Weibull distribution, which turns out to generate a lower Akaike Information Criterion than other distributions. Results (column III) are qualitatively the same as those obtained from the semi-parametric estimation.

## Discussion

The analysis in this paper delivers two key findings. First, a deeper tradition of nanny statism increases the likelihood of early adoption of CCM, but it does not seem to affect the stringency of the measures adopted. Second, early adoption of CCM significantly reduces the death toll from the first wave of COVID-19. These two findings can be interpreted in light of the hypothesis advanced in Sect. 1. A deeper tradition of nanny statism influences the attitude of both incumbent government and citizens towards paternalist interventions and regulations. This in turn makes it more likely that the response to a new and unknown threat will take a paternalist form, irrespective of other factors that might contribute to shaping this response, such as the degree of democracy of the country, the ideology of the incumbent, etc…In other words, past experiences of nanny statism call for more nanny statism when facing new threats. To the extent that the government effectively “knows better”, this effect translates into an improvement in public health outcomes. Note, that the central tenement of the hypothesis of the paper is about the first of these two findings, i.e. the effect of a tradition of nanny statism on the nature of the response to the new challenge. The second finding is a corollary to the main hypothesis.

From a public policy perspective, the paper contributes to the debate on the desirability of paternalism. This debate has primarily focused on whether a paternalist norm (or set of norms) directly affects behaviour and/or outcomes that are specifically relevant to that norm, e.g. whether norms that limit the use of smoking or alcohol reduce the prevalence of smoking or drinking and/or associated illnesses. This paper goes one step further and argues that the adoption of paternalist measures has an impact on country’s culture. When a government adopts a norm for, say, mandatory helmets or seatbelts, societal attitudes are affected in such a way that it then becomes easier and more acceptable for future governments to adopt paternalist measures in response to new public health threats that are unrelated to safety on bikes, motorbikes, or cars. The corollary is that these new measures will produce better health outcomes only to the extent that the government knows better, which indeed seems to be the case in the empirical context of this paper. Therefore, the accumulation of paternalist norms over time has a cultural effect that potentially benefits the society above and beyond the specific risk factors that the norms are meant to moderate.

There remains the question of why nanny statism affects the likelihood of early adoption of CCM, but not their level of stringency. A possible answer revolves around compliance. An ancillary result of this paper is that there is evidence of a positive effect of nanny statism on citizen’s compliance with CCM. While this bit of evidence requires some more investigation, it does point to a potentially interesting mechanism: in a paternalist country, the government intervenes earlier and people comply more, which in turn increases the effectiveness of government intervention and reduces the need for more draconian measures later. A full test of this mechanism would require the estimation of a structural system of equations and is therefore proposed as an avenue of future work.

While COVID offers a suitable scenario to test the hypothesis, some limitations of this analysis should also be acknowledged. From a statistical perspective, a valid test of the hypothesis requires that other factors affecting the decision of the government (or the death toll) are properly considered. Since there is no consensus in the literature on what these other factors might be, one has to proceed pragmatically and the selection of control variables in the regression models inevitably involves some degree of subjectivity. The sensitivity analysis presented in this paper addresses this concern, at least to some extent. Also, early in 2020, several international experts and institutions provided recommendations regarding the adoption of CCM. Therefore, it cannot be excluded that some countries adopted CCM in response to this advice and/or to mimic other countries, irrespective of their tradition of paternalism. If this were the case, then the statistical significance of the estimated coefficient of the nanny state index might be reduced. The choice to measure CCM stringency as of 1 March 2020 attenuates the problem. Also, the paper has produced some evidence that the role of nanny statism is robust to the inclusion of spatial effects. However, if one could entirely exclude this “mimicking effect”, then the statistical strength of the estimated coefficient of the nanny state index would probably increase.

## Conclusions

To the best of the author’s knowledge, this is the first paper that provides a conceptual framework and some empirical evidence of a broader cultural effect of paternalism. This effect is potentially significant in terms of achieving better public health outcomes when new and unknown threats emerge. These considerations lead to some avenues of future research.

In particular, it would be interesting to think of alternative scenarios for the test of the hypothesis. One that would appear to be particularly promising involves the response to natural disasters. Full information about the nature of natural hazards is often missing, which in a sense provides a justification to public interventions. At the same time, these interventions might not only be contrary to immediate individual preferences, but also involve additional costs borne either by private individuals or the community. This then poses a dilemma, or at least a trade-off, that different countries are likely to address in different ways. The hypothesis of this paper would suggest that more pervasive government regulation to mitigate natural disasters is likely to be observed if the state has been more of a nanny in the past. To the extent that this regulation effectively reduces hazards, then one should also observe that higher values of the nanny state index reduce the cost (monetary and human) of natural disasters, when these happen. More generally, the relationship between nanny statism and the incidence and cost of natural disasters is a research question that deserves future attention.

There are also several avenues of further research that specifically relate to the response to COVID-19. Some of these have been flagged throughout the paper. In particular, the analysis of hazard ratios can be extended to incorporate time varying factors and/or to shed further light on how paternalism affects the intensity of CCM stringency; the links between paternalism, early adoption of CCM, compliance, and public health outcomes should be further investigated using a system of equations with panel data, and alternative spatial specifications could be used to test the interdependencies of decision-making across neighbouring countries. Lastly, public health outcomes other than the death toll from COVID-19 should be considered in the analysis.

### Electronic supplementary material

Below is the link to the electronic supplementary material.

Supplementary Material 1

## Data Availability

The data used are described in the Appendix in the Supplementary Materials and are all from publicly available datasources; links are provided in the Appendix. The full dataset and codes can be obtained from the author and can be published together with the paper.
