# Supplementary material for: Why nanny statism matters: evidence from the first wave of COVID-19
Source: BMC Public Health. 2024 Jul 23;24:1963. doi: 10.1186/s12889-024-19477-8 (PMC11265364; doi:10.1186/s12889-024-19477-8)
Supplement: Supplementary file 1 — Supplementary Material 1 [file 12889_2024_19477_MOESM1_ESM.docx]

**Supplementary Materials**

*The nanny state index*

The index here used to measure the nanny state is constructed as follows. Let *i* be the generic country and *j* a generic category of potential government intervention. Also let *J* be the total number of categories. Define *D*_i,j_ a dummy variable such that

$$D_{i,j}=\left\{ \begin{matrix} 1 \\ 0 \end{matrix} \right.\begin{matrix} if country i 1 adopts a law or regulation in category j \\ \mathrm{otherwise} \end{matrix}$$

The index for country *i* is then defined as the average of *D’s* taken across all *j’s* :

$$nanny state index=\frac{\sum_{j=1}^{J} D_{i,j}}{J}$$

The total number of categories J is normally 21. However, for some countries, data on some categories are not available. In these cases, J is set equal to the number of categories for which data are available. In words, the nanny state index counts the proportion of categories in which the government intervenes with a low, regulation or policy that restricts or affects the free choice/behaviour of consumers/individuals.

The 21 categories and an example of the computation of the index are illustrated in the table below:

| **Categories** | **D_Argentina,j_** |
| --- | --- |
| Existence of a national child-restraint law | 1 |
| Law requires helmet to be fastened (motorbike) | 1 |
| Existence of a national seat-belt law | 1 |
| Country (vehicle standards) - summary index | 1 |
| Existence of any policies on marketing of foods to children | 0 |
| Existence of any policies to reduce population salt consumption | 1 |
| Existence of national policies on saturated fatty acids | 1 |
| Existence of national policies on trans-fatty acid elimination | 1 |
| Existence of operational policy/strategy/action plan to reduce unhealthy diet related to NCDs | 1 |
| Existence of operational policy/strategy/action plan to reduce the harmful use of alcohol | 1 |
| Existence of operational policy/strategy/action plan to reduce physical inactivity | 1 |
| Implementation of physical activity public awareness program | 1 |
| Health warning labels on alcohol advertising | 1 |
| Health warning labels on alcohol containers | 1 |
| Health warning labels on drink-driving | 0 |
| Health warning labels on pregnancy | 0 |
| Health warning labels on under-age drinking | 0 |
| Number of standard drinks reported on beverage containers | 0 |
| On premise restriction of sales to intoxicated persons) | 0 |
| Restriction on alcohol use in public places | 1 |
| Legally binding lead controls | 1 |
| **Index of nanny state for Argentina** | **15/21 = 0.71** |

In Argentina, there are laws that enforce child restraint in cars, hence the dummy variable for the category “existence of national child-restraint law” is 1. Conversely, there are no laws or regulations that require the number of standard drinks to be reported on beverage containers, hence the dummy variable for the category “number of standard drinks reported on beverage containers” is 0. In total, there are 15 categories coded as 1 out of a total of 21, so the index of nanny state for Argentina is 15/21 = 0.71.

This approach to measuring the nanny state has admittedly two limitations. The first one is that the binary variables provide a rather crude representation of the depth and extent of each law or policy intervention. On the other hand, a more granular coding of the index would require more detailed information than what is available for most countries, and a more subjective assessment, which would in turn raises questions of comparability and reliability.

The second limitation relates to the fact that index is calculated as a simple average of 21 selected categories. In other words, all the 21 selected categories have the same weight and other categories that could be potentially included have instead a zero weight. A possible way to extend the index is to code an even larger set of categories and then use some form of factor analysis to reduce the dimensionality of the dataset.

While this is certainly an avenue for future research, some sensitivity analysis is conducted here by expanding the dataset to include four categories that relate to smoking-free legislation and bans. These are not included in the original set of 21 categories to minimise the risk of spurious correlations in estimating the effect of nanny statism. In fact, if smoking bans affect smoking prevalence and smoking prevalence affects the COVID death toll and/or the perception of population’s vulnerability to COVID, then the estimated coefficient of the nanny state index in the regressions of Tables 1 and 2 of the paper would probably overestimate the impact of paternalism. The four categories added for the purpose of the sensitivity analysis are

| National smoke free legislation |
| --- |
| ban on tobacco advertising |
| warning on damages from tobacco |
| taxes on tobacco |

The index including these four new categories is highly correlated (0.95) with the index based on the 21 original categories. Not surprisingly, results of the regression analysis are qualitatively unchanged, as shown in columns III and IV of Table 2 of the paper.

*Table A: Additional sensitivity analysis*

|  | I  Stringency index | II  Stringency index > 0 | III  Stringency index | IV  Stringency index | V  Stringency index | VI  Stringency index | VII  Stringency  Dummy | VIII  Stringency  Dummy | IX  Stringency  Index | X  Stringency Index > 0 |
| --- | --- | --- | --- | --- | --- | --- | --- | --- | --- | --- |
| Nanny state | 7.515  (5.001) | -3.449  (5.291) | 1.683  (4.422) | 3.470  (6.157) | 10.096*  (5.935) | 6.827  (4.932) | 2.855***  (1.051) | 2.243***  (.851) | 6.197  (4.880) | -4.100  (5.654) |
| GDPpc | 1.461  (1.386) | 1.174  (1.273) | 4.342**  (1.657) | -.436  (1.612) | -1.163  (2.146) | 1.521  (1.497) | .061  (.235) | .186  (.193) | .327  (1.283) | -.141  (1.299) |
| Population density | .005  (.005) | -.000  (.005) | .002**  (.001) | .006  (.004) | .009**  (.004) | .004  (.005) | .002  (.001) | .001  (.001) | .005  (.004) | .000  (.004) |
| Elderly Pop. share | -715**  (.321) | -.500  (.312) | -.144  (.348) | -.852**  (.397) | -.350  (.268) | -.651**  (.327) | -.078*  (.046) | -.072**  (.029) | -.471*  (.257) | -.241  (.227) |
| Government effectiveness | .674  (2.211) | 3.111  (2.495) | -3.911  (2.755) | 1.768  (2.371) | 1.871  (3.727) | .944  (2.375) | -.175  (.347) | -.151  (.227) | .580  (2.192) | 2.224  (2.615) |
| Polity | .580*  (.322) | .423  (.321) | .552**  (.251) | .562*  (.288) | .109  (.433) | .593*  (.343) | .066**  (.031) | .084***  (.026) | .577**  (.290) | .451  (.336) |
| Distance from China | -6.809*  (4.040) | -7.114  (4.670) | -8.978***  (1.099) | -7.724*  (4.182) | 1.612  (3.083) | -7.084*  (4.147) | -.345  (.262) | -.490**  (.237) | -6.056*  (3.298) | -6.005  (3.835) |
| Obesity |  |  |  | .213  (.170) |  |  |  |  |  |  |
| Diabetes |  |  |  | -.060  (.291) |  |  |  |  |  |  |
| TBC |  |  |  | .005  (.007) |  |  |  |  |  |  |
| Political ideology |  |  |  |  | -1.187  (1.118) |  |  |  |  |  |
| COVID-19 cases end of Feb 2020 |  |  |  |  |  | -1.273***  (.429) |  |  |  |  |
| Affected by SARS or MARS |  |  |  |  |  |  | .324  (.779) | .171  (.366) | 8.282  (6.164) | 8.324  (6.973) |
| Affected by Ebola |  |  |  |  |  |  | .729  (.579) | .764  (.532) | 1.347  (3.778) | -1.327  (4.451) |
| Constant | 55.125*  (31.586) | 69.500*  (38.205) | 48.696***  (14.342) | 73.567*  (38.766) | .019  (25.517) | 57.429*  (31.326) | 1.660  (3.111) | 2.166  (2.690) | 56.338*  (28.655) | 68.466  (34.002) |
| Observations | 92 | 59 | 146 | 92 | 42 | 91 | 92 | 146 | 92 | 59 |

Note: This table presents some sensitivity analysis that complements the results reported in Table 3 of the paper. Columns I to VI show estimates of the extended specification of the linear regression model, with the full numerical stringency index (i.e. the index that takes value from 0 to 100) as the dependent variable. Columns VII and VIII present estimates from an extended specification of the probit regression which controls for past history of zoonotic diseases (SARS, MARS and Ebola) and natural disasters. Columns IX and X presents linear regression estimates from the same specifications with controls for zoonotic diseases; the dependent variable in these two columns is the full numerical stringency index. In Columns I, IV, V, VI, VII, IX and X the sample is restricted to countries that had not yet reported a COVID-death by end of February 2020. In Column II and X the sample is further restricted to only include strictly positive values of the stringency index. In Columns III and VIII, all available observations are used for estimation. *, **, *** denote statistical significance at 10%, 5%, and 1% confidence level, respectively.

*Table B: Full set of estimates for Table 4 of the paper*

|  | I | II | III | IV | V | VI | VII | VIII | IX | X | XI | XII | XIII |
| --- | --- | --- | --- | --- | --- | --- | --- | --- | --- | --- | --- | --- | --- |
|  | Mobility | Mobility | Mobility | Mobility | Mobility | Mobility | Mobility | Mobility | Mobility | Stringency dummy | Stringency dummy | Stringency dummy | Stringency dummy |
| Nanny state | 24.651***  (6.786) | 26.409***  (8.399) | 34.783***  (9.048) | 23.490***  (7.379) | 26.867**  (13.468) | 28.614***  (7.582) | 28.855***  (7.742) | 16.364*  (9.658) | 18.425*  (10.003) | 2.447***  (.953) | 2.545***  (.991) | 2.430**  (.983) | 2.327**  (.975) |
| COVID death toll  (end of March 2020) | .284***  (.051) | .324***  (.054) | 1.419***  (.356) | 1.300***  (.276) | .324***  (.063) | .234***  (.047) | .238***  (.046) | .253***  (.054) | .257***  (.052) |  |  |  |  |
| Stringency index  (end of March 2020) | .801***  (.114) |  |  | .738***  (.173) |  | .747***  (.104) | .505***  (.247) | .511**  (.204) | .153  (.410) |  |  |  |  |
| Trust in people |  |  |  |  | -53.537***  (16.902) | -.19.052  (14.116) | -76.546  (56.895) |  |  |  |  |  |  |
| Trust in government |  |  |  |  |  |  |  | -65.753  (51.522) |  |  |  |  |  |
| Trust in neighbours |  |  |  |  |  |  |  |  | -88.630*  (48.890) |  |  |  |  |
| Trust*Stringency Index |  |  |  |  |  |  | .719  (.745) | .674  (.649) | .779  (.580) |  |  |  |  |
| GDPpc |  |  |  |  |  |  |  |  |  | .170  (.395) | .188  (.197) | .204  (.200) | .187  (.196) |
| Pop. Density |  |  |  |  |  |  |  |  |  | .001  (.001) | .002  (.001) | .002  (.001) | .002  (.001) |
| Elderly population |  |  |  |  |  |  |  |  |  | -.051  (.170) | -.045  (.037) | -.055  (.038) | -.053  (.037) |
| Gov effectiveness |  |  |  |  |  |  |  |  |  | -.159  (.308) | -.172  (.303) | -.131  (.300) | -.134  (.301) |
| Stringency in neighbour countries (binary) |  |  |  |  |  |  |  |  |  | -.247  (.326) |  |  |  |
| Stringency in neighbour countries (value) |  |  |  |  |  |  |  |  |  |  | -.022  (.018) |  |  |
| COVID deaths in neighbour countries (binary) |  |  |  |  |  |  |  |  |  |  |  | -.301  (.444) |  |
| COVID deaths in neighbour countries (value) |  |  |  |  |  |  |  |  |  |  |  |  | -.044  (.287) |
| constant | -23.799**  (11.224) | 37.752  (4.626) | 32.657***  (4.755) | -20.281  (15.191) | 51.276***  (8.140) | -16.140  (11.346) | 3.713  (21.377) | 10.893  (18.137) | 54.390*  (32.430) | -1.798  (.1879( | -2.070  (1.838) | -2.203  (1.870) | -2.060  (1.832) |
| N. of obs. | 123 | 123 | 77 | 73 | 55 | 54 | 54 | 67 | 66 | 99 | 99 | 99 | 99 |

Notes: : In rows I to IX estimation is by linear regression. The dependent variable is the index of mobility as of 1 April 2020; an increase in the index denotes greater compliance with CCM. The interactive terms Trust*Stringency is constructed using trust in people in column VII, trust in government in column VIII and trust in neighbours in column IX. In rows X to XIII estimation is by probit regression and the dependent variable takes value 1 if a country had adopted any CCM as of 1 March 2020. The full sample includes all countries for which data are available. The restricted sample includes only countries that had not yet reported a COVID death as of end of February 2020 and for which other data are available. This restricted sample is used for consistency with the regressions reported in Tables 2 and 3. Robust standard errors are reported in brackets. *, **, *** denote statistical significance at 10%, 5%, and 1% confidence level, respectively

*Table C Full set of estimates for Table 5 of the paper*

|  | I | II | III | IV | V |
| --- | --- | --- | --- | --- | --- |
| Nanny state index | 1.028  (.001) | 1.025  (.001) | 1.024  (.001) | 1.014  (.030) | 1.013  (.011) |
| GDP p.c. | 1.119  (.545) | 1.249  (.165) | 1.200  (.268) | 1.103  (.526) | 1.167  (.257) |
| Population density | 1.002  (.001) | 1.002)  (.034) | 1.002  (.002) | 1.001  (.001) | 1.001  (.001) |
| Elderly population | .937  (.066) | .963  (.241) | .953  (.134) | .959  (.099) | .968  (.156) |
| Government effectiveness | .907  (.751) | .701  (.161) | .773  (.298) | 1.130  (.547) | ,999  (.996) |
|  |  |  |  |  |  |
| Observations | 98 | 98 | 98 | 152 | 152 |
| Test of PH assumption  (p-value) | 3.97  (.5534) | 9.49  (.0911) | … | 5.91  (.3152) | 5.34  (.3759) |
| Start date | 1 Jan 2020 | 1 Jan 2020 | 1 Jan 2020 | 1 Jan 2020 | 1 Jan 2020 |
| End date | Day of adoption | Day of adoption | Day of adoption | Day of adoption | Day of adoption |
| Censoring | Yes | No | No | Yes | No |
| Sample | Restricted | Restricted | Restricted | Full | Full |

Notes: The table reports the hazard ratios from the Cox Proportional Hazard model, except form Column III where hazard ratios are estimated from a parametric model based on the Weibull distribution. P-values for the test of the null hypothesis that the hazard ratio is 1 are reported in brackets. The test of the proportional hazard (PH) assumption is a chi-square test of the null hypothesis that the log hazard-ratio function is constant over time; that is, that the hazard ratio is constant over time. The table reports the value of the chi-square test statistic and the corresponding p-value in brackets. In some models, countries that had not yet adopted CCM as of 1 March 2020 are treated as censored observations (Columns I and IV). The restricted sample includes only countries that had not yet reported a COVID death as of end of February 2020. The full sample includes all countries for which data are available. The set of controls is the same as in Table 2.

*Variables description, sources, and summary statistics*

| Name | Definition | Source | Average  restricted sample | Std dev. restricted sample | Average  full sample | Std dev.  full sample |
| --- | --- | --- | --- | --- | --- | --- |
| Nanny State | Extent to which a country adopts policies that substitute for personal choice. Measured by the proportion of such policies (out of a sample of 21 categories) adopted by the government as of end of 2019 | Calculated from data in WHO Global Health Data Repository  <https://apps.who.int/gho/data/view.main.51417> | .405 | .209 | .465 | .216 |
| Nanny stateextended | Extended version of the nanny state index based on 25 categories. | Calculated from data in WHO Global Health Data Repository  <https://apps.who.int/gho/data/view.main.51417> | .426 | .227 | .488 | .230 |
| Stringency dummy | Dummy variable that takes value 1 if the stringency index (see below) is strictly positive as of 1 March 2020 and 0 otherwise | Constructed from the data in Hale et al. (2020)  <https://www.bsg.ox.ac.uk/research/research-projects/coronavirus-government-response-tracker> | .634 | .483 | .707 | .456 |
| Stringency index | Index of the strictness of “lockdown style” policies that primarily restrict people’s behaviour. The index takes value 0 if no such policies are in place as of 1 March 2020. Higher values denote stricter policies. | Hale et al. (2020)  <https://www.bsg.ox.ac.uk/research/research-projects/coronavirus-government-response-tracker> | 7.593 | 8.862 | 11.419 | 13.155 |
| Stringency index > 0 | A version of the stringency index that only includes strictly positive values as of 1 March 2020 | Constructed from the data in Hale et al. (2020)  <https://www.bsg.ox.ac.uk/research/research-projects/coronavirus-government-response-tracker> | 11.961 | 8.448 | 16.138 | 12.976 |
| GDPpc | Natural logarithm of GDP per capita in constant US dollars | Calculated from data available from World Bank Development Indicators <https://databank.worldbank.org/source/world-development-indicators> | 8.946 | 1.185 | 9.281 | 1.207 |
| Population density | Population per square meter | World Bank Development Indicators  <https://databank.worldbank.org/source/world-development-indicators> | 191.213 | 383.111 | 328.162 | 1507.857 |
| Elderly population share | Share of population aged 65 or older | World Bank Development Indicators  <https://databank.worldbank.org/source/world-development-indicators> | 7.219 | 5.188 | 8.808 | 6.205 |
| Government effectiveness | Index of quality of public services, capacity of the civil service and its independence from political pressures, and the quality of policy formulation | World Governance Indicators  <https://info.worldbank.org/governance/wgi/> | -.242 | .889 | -.002 | 1.000 |
| Polity | Quality of the polity defined in terms of competitiveness and openness of executive recruitment, constraint on chief executive, and competitiveness of political participation, defined on a scale from -10 to +10, where -10 denotes a perfect autocracy. | Polity Database  https://www.systemicpeace.org/polityproject.html | 4.067 | 5.829 | 4.345 | 6.092 |
| Hospital beds | Hospital beds for 1,000 people | World Bank Development Indicators  <https://databank.worldbank.org/source/world-development-indicators> | 2.596 | 1.960 | 3.089 | 2.522 |
| Obesity | Percentage of adult population with Body Mass Index greater than or equal to 25 | WHO Global Health Data Repository  <https://apps.who.int/gho/data/view.main.BMI25Cv> | 44.103 | 16.965 | 46.890 | 17.372 |
| Diabetes | Percentage of people ages 20-79 who have type 1 or 2 diabetes | Our World in Data  <https://ourworldindata.org/grapher/diabetes-prevalence> | 8.116 | 4.500 | 8.061 | 4.256 |
| TBC | Incidence of tuberculosis per 100,000 people | World Bank Development Indicators  <https://databank.worldbank.org/source/world-development-indicators> | 115.360 | 143.909 | 98.358 | 134.212 |
| Political Ideology | Ideological location of the ruling party, 1 = right, 0 = centre, -1 = left. Big tent parties and broad coalitions spanning across the ideological spectrum are generally classified as 0. Divided governments are also classified as 0. | Database of Political institutions  <https://publications.iadb.org/en/database-political-institutions-2020-dpi2020> | .906 | .904 | 1 | .918 |
| Distance from China | Natural logarithm of geographical distance between a country and China | Calculated from distances provided in Google Maps | 9.094 | .507 | 8.925 | .821 |
| COVID death toll | Natural logarithm of COVID-related deaths per million people | Calculated from data in Our World in Data  <https://ourworldindata.org/covid-deaths> | 2.421 | 1.901 | 2.769 | 1.987 |
| COVID cases as of end of February 2020 | Number of cases per million people reported on 29 February 2020 (average of preceding week) | WHO COVID-19 Dashboard accessed via Our World in Data  <https://ourworldindata.org/covid-cases> | 9.526 | 101.379 | 8.488 | 86.262 |
| Mobility | Absolute value of the percentage change in the number of retail and recreation visitors relative to before the pandemic, as of 1 April 2020. | Google COVID-19 Community Mobility Trends  <https://www.google.com/covid19/mobility/> | 51.177 | 22.402 | 53.100 | 22.480 |
| Stringency in neighbour countries (Binary) | Dummy variable that takes value 1 if the stringency index as of 1 March 2020 is strictly positive in at least one neighbouring country. Countries are considered neighbouring if they share a land border | Constructed from the data in Hale et al. (2020)  <https://www.bsg.ox.ac.uk/research/research-projects/coronavirus-government-response-tracker> | .563 | .497 | .602 | .490 |
| Stringency in neighbour countries (Value) | Average value of the stringency index in neighbouring countries as of 1 March 2020. Countries are considered neighbouring if they share a land border. | Constructed from the data in Hale et al. (2020)  <https://www.bsg.ox.ac.uk/research/research-projects/coronavirus-government-response-tracker> | 5.640 | 7.420 | 7.107 | 9.208 |
| COVID deaths in neighbour countries (Binary) | Dummy variable that takes value 1 if the COVID death toll as of 1 March 2020 at least one neighbouring country. Countries are considered neighbouring if they share a land border. | Constructed from data in Our World in Data  <https://ourworldindata.org/covid-deaths> | .106 | .309 | .167 | .374 |
| COVID deaths in neighbour countries (Values) | Total COVID death toll per million people in neighbouring countries as of 1 March 2020. Countries are considered neighbouring if they share a land border | Constructed from data in Our World in Data  <https://ourworldindata.org/covid-deaths> | .108 | .309 | .160 | .519 |
| Trust in people | Share of people agreeing with the statement "most people can be trusted" | Integrated Values Survey, 2022 | .181 | .114 | .249 | .160 |
| Trust in government | Share of people that responded “a great deal” or “quite a lor” to the question of how much confidence they have in their national government. | Constructed from indicator e069_11 in the World Values Survey 7. Only years prior to 2020 are included  <https://www.worldvaluessurvey.org/WVSDocumentationWV7.jsp> | .155 | .073 | .248 | .181 |
| Trust in neighbours | Share of people that responded “completely” or “somewhat” to the question of whether they trust their neighbours | Coded from indicator g007_18_b in the World Values Survey 7  Only years prior to 2020 are included  <https://www.worldvaluessurvey.org/WVSDocumentationWV7.jsp> | .703 | .139 | .751 | .135 |
| Affected by SARS or MARS | Dummy variable that takes value 1 if a country was affected by SARS or MARS | Coded from information available from WHO at <https://www.who.int/publications/m/item/summary-of-probable-sars-cases-with-onset-of-illness-from-1-november-2002-to-31-july-2003>  and  <https://www.who.int/news-room/fact-sheets/detail/middle-east-respiratory-syndrome-coronavirus-(mers-cov)> | .059 | .237 | .196 | .398 |
| Affected by Ebola | Dummy variable that takes value 1 if a country was affected by Ebola | Coded from information available from <https://www.gov.uk/government/publications/ebola-origins-reservoirs-transmission-and-guidelines/ebola-overview-history-origins-and-transmission#> | 0.53 | .224 | .047 | .214 |

Note: average and standard deviation for each variable are calculated on two sample: (i) the restricted sample of countries that had not yet reported a COVID death as of end of February 2020 and (ii) the full sample of all countries for which information on that variable is available. The restricted sample is the one primarily used in the paper. In the baseline specification it consists of 99 observations, albeit in some extended specifications the number of observations is reduced due to lack of data for some countries. The size of the full sample instead varies depending on the specific variable.
